# Unleashing rapamycin in fibrosis

**DOI:** 10.18632/oncotarget.4652

**Published:** 2015-06-26

**Authors:** Alexander T. Hillel, Alexander Gelbard

**Affiliations:** Department of Otolaryngology - Head and Neck Surgery, Johns Hopkins University School of Medicine, Baltimore, Maryland, USA

Rapamycin (sirolimus, rapamine) is a macrocyclic antibiotic produced by the bacteria *streptomyces hygroscopicus*, with immunosuppressive and antiproliferative capabilities. Rapamycin binds with FK- binding protein 12 to inhibit the mechanistic (formerly mammalian) Target of Rapamycin (mTOR) protein [1]. Our laboratory recently demonstrated antifibroblast effects with rapamycin treatment in vitro [2]. We derived pathologic fibroblasts from patients with laryngotracheal stenosis (LTS) and demonstrated reduced fibroblast proliferation, collagen expression, and cellular metabolism with rapamycin treatment. LTS is the clinical disease of fibrosis of the larynx and trachea and may be used as a model of fibrosis in other organs. Over the course of this editorial we will discuss rapamycin's therapeutic rationale and potential for benign diseases such as fibrosis.

Fibrosis is the terminal pathological outcome of myriad chronic inflammatory diseases. The process is defined by the excessive accumulation of fibrous connective tissue components of the extracellular matrix (ECM, i.e. collagen and fibronectin) in inflamed tissue [3]. The excessive collagen deposition ultimately results in organ-specific injury, which in LTS manifests as airway obstruction and clinical dyspnea. While fibroblasts are thought to represent the end effector cell in LTS, by the time increased collagen deposition results in clinical stenosis of the larynx and trachea it is too late to effectively intervene medically, committing these patients to repeated surgical dilation and/or excision of fibrosis with the morbidity associated with invasive surgical procedures. In another study by our laboratory, we demonstrated scar fibroblasts were highly proliferative compared to normal fibroblasts yet used the inefficient metabolic process of aerobic glycolysis to preferentially drive hyperplasia to form scar (unpublished). This phenomenon is similar to the Warburg effect where cancer cells drive rapid growth through an extremely high rate of aerobic glycolysis. While the Warburg-like effect seen in LTS fibroblasts *in vitro* is less pronounced than the Warburg effect in cancer, identifying the cellular metabolism behind fibrosis suggests at potential therapies directed at fibroplasia. Our investigations into fibroblast metabolism and rapamycin's inhibitory effect suggests at a critical link between cellular metabolism and unregulated fibroblast proliferation that could elucidate rational druggable targets to mitigate LTS and fibrosis in general.

mTOR's regulatory role makes the protein a critical node between metabolism and cell proliferation. Environmental factors such as stress and energy influence mTOR, which in turn regulates protein, lipid, and nucleotide biosynthesis, mitochondrial proliferation and function, cell growth and development amongst other cell functions. In fibroblasts mTOR regulates cell kinetics and collagen and other extracellular matrix molecule production. Inhibition of mTOR has been explored in cancer, cardiovascular disease, autoimmunity, and metabolic disorders. In immune cells including T-lymphocytes and macrophages, mTOR subunit inhibition results in reduced metabolism which in turn impacts immune cell activation and function [4]. Based on the results of our studies, we hypothesize that mTOR has a significant role in abnormal fibroblast proliferation and collagen synthesis, which should allow for modulation of the mTOR mechanism with rapamycin or other rapalogs.

Alternatively myofibroblasts have been the focus of extensive research in fibrosis, given their role in secreting ECM and collagen along with their fundamental contribution to wound contracture. Epithelial-mesenchymal transition (EMT), a process whereby fully differentiated epithelial cells undergo transition to a mesenchymal phenotype giving rise to myofibroblasts. EMT is increasingly recognized as a contributing factor to tissue fibrosis following epithelial injury and a fundamental process involved in carcinogenesis and metastasis. Inhibition of mTOR signaling through rapamycin has been shown to attenuate the process of EMT. This offers additional mechanistic rationale for mTOR inhibition as a therapeutic strategy in human fibrosis.

Strong preclinical and human evidence also offers support for the use of rapalogs beyond its effects modulating the end-effectors of fibrinogenesis (fibroblasts & myofibroblasts). In preclinical models, immunocompromised mice exhibit decreased fibrosis during wound repair [5]. T-cell signaling appears to be a large contributor to fibrinogenesis across multiple organ systems with T-cell-mediated mechanisms key regulators of fibrotic repair. Interestingly rapalogs are utilized clinically in the transplant setting for T-cell inhibition and clinical immunosuppression.

Rapamycin is a Food and Drug Administration (FDA)-approved drug that promotes long term tolerance in organ transplant patients, and is incorporated within coronary stents to reduce restenosis and accelerated arteriopathy. Rapamycin and rapalogs mechanism of action is to bind to FKBP12 to inhibit a subunit protein of mTOR, mTORC1 [1]. Prolonged exposure of rapamycin has been shown to reduce activity in the other subunit, mTORC2, as well. A few studies in addition to our own demonstrated rapamycin to mitigate aberrant wound healing and fibrosis in other organ systems, presumed to be through mTOR-mediated immunosuppressive and antifibroblast effects [2, 6, 7]. Figure 1 illustrates the proposed mechanism by which rapamycin inhibits fibroblast hyperplasia and hypertrophy as well as inflammation in LTS. The immunosuppressive and antifibroblast effects suggest at the potential of rapamycin treatment as adjuvant therapy for LTS. Modulating the immune and fibroblast response should be more effective than current medical therapies.

**Figure 1:**
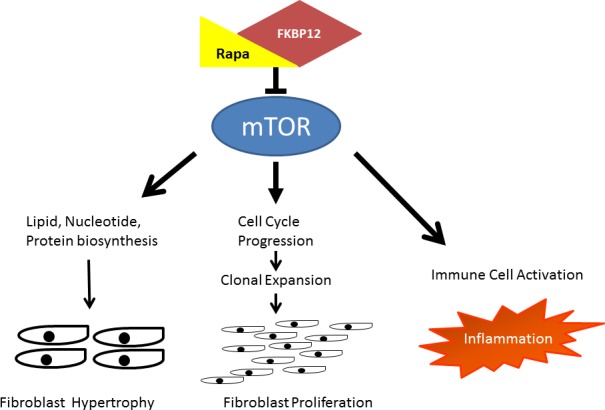
Proposed schematic of rapamycin inhibition of fibrosis Rapamycin binds with FK-binding protein 12 to inhibit the mechanistic Target of Rapamycin (mTOR). Proposed downstream effects of mTOR inhibition on fibroblasts include reduced lipid, nucleotide, and protein biosynthesis which results in decreased size and cell cycle inhibition which results in decreased proliferation. Furthermore, suppression of immune cells and their mediators attenuates inflammation and its resultant stimulatory effect on fibroblasts.

Outstanding questions to be addressed include the specific signaling pathway by which rapamycin inhibits fibroplasia, and if more specific targeting of these cellular mechanisms would be effective in treating the disease in the trachea as well as other organs. Future studies will also address therapeutic delivery with sustained topical application ideal to address organ specific fibrosis and avoid side effects associated with systemic administration. Specific to LTS, a rapamycin-eluting stent could assist in suppressing the inflammatory response, fibroblast proliferation, and collagen synthesis seen in LTS while providing structural support of the tracheal wall.

## References

[1] Saunders RN (2001). Kidney Int.

[2] Namba DR (2015). Otolaryngol Head Neck Surg.

[3] Wynn TA (2012). Nat Med.

[4] Powell JD (2012). Annu Rev Immunol.

[5] Ghosh A (2011). Otolaryngol Head Neck Surg.

[6] Chen G (2012). PLoS One.

[7] Yu SY (2013). Thorac Cardiovasc Surg.

